# Intraocular Pressure Changes after Water Drinking Test in Surgically Treated Primary Congenital Glaucoma

**DOI:** 10.18502/jovr.v15i3.7450

**Published:** 2020-07-29

**Authors:** Reza Razeghinejad, Zahra Tajbakhsh, Masoumeh Beigom Masoumpour, M. Hossein Nowroozzadeh

**Affiliations:** ^1^Glaucoma Service, Wills Eye Hospital, Philadelphia, PA, USA; ^2^Poostchi Ophthalmology Research Center, Shiraz University of Medical Sciences, Shiraz, Iran

**Keywords:** Glaucoma Drainage Device, Intraocular Pressure, Primary Congenital Glaucoma, Trabeculotomy, Water Drinking Test

## Abstract

**Purpose:**

To assess intraocular pressure (IOP) changes after the water drinking test (WDT) in patients with primary congenital glaucoma (PCG).

**Methods:**

In this prospective interventional study, 20 eyes of 20 patients with PCG were included. All patients had undergone trabeculotomy. Six out of twenty eyes had received a glaucoma drainage device (GDD) implantation. IOP was measured using an air-puff tonometer at baseline, and 15, 30, 45, and 60 min after WDT. The repeated-measures analysis of variance test was used to compare the mean IOPs at different time points.

**Results:**

The mean (± standard deviation) of participants' age was 9.9 ± 2.7 years (range, 6 to 16 years), and 8 (40%) participants were male. The mean IOPs at baseline and 15, 30, 45, and 60 minutes after the WDT were 15.8 ± 3.7, 18.6 ± 3.4, 19.0 ± 3.8, 17.9 ± 3.8, and 16.9 ± 3.5 mmHg, respectively (*P* < 0.001). Pairwise comparisons revealed that the mean IOPs after 15 and 30 min were significantly greater than the baseline IOP (*P* < 0.001 and *P* = 0.002, respectively); however, the difference in mean IOPs after 45 and 60 min were not statistically significant from the baseline IOP. The averages of IOP peak and IOP fluctuation after the WDT were 20.0 ± 3.5 and 4.2 ± 2.9 mmHg, respectively. IOP fluctuation in those who underwent trabeculotomy alone was twice that of those with GDDs, but the difference was not statistically significant (5.0 vs 2.5 mmHg; *P* = 0.08).

**Conclusion:**

In patients with PCG, WDT induced significant IOP elevation 15 and 30 min after the test, which returned to pre-test values after 45 min.

##  INTRODUCTION

Primary congenital glaucoma (PCG) is the most common hereditary type of glaucoma in childhood.^[1]^ Several mechanisms have been suggested for the development of PCG, which result in angle dysgenesis and compromise outflow through the trabecular meshwork. Goniotomy and trabeculotomy have been recommended as the initial procedures to improve outflow by removing the abnormal trabecular tissue and making a direct connection between the anterior chamber and the Schlemm's canal. Trabeculectomy and glaucoma drainage device (GDD) implantation are employed if the intraocular pressure (IOP) cannot be controlled with the aforementioned procedures or glaucoma medications.^[2]^ The goal of glaucoma medical and surgical interventions is to keep the IOP at a specific level in order to halt or slow down glaucoma progression.^[3]^

IOP is a dynamic parameter with an individual circadian rhythm. Currently, management of glaucoma include IOP measurements during clinic hours performed a few times a year. A diurnal curve may be used to evaluate glaucoma progression in a patient when the office IOP is within an acceptable range. The most common methods for assessing the diurnal curve in glaucoma patients are IOP readings at different time points during clinic hours and hospitalization in a sleep laboratory; both are cumbersome and costly. The Water Drinking Test (WDT) has been suggested as a practical and easy-to-perform test to estimate the diurnal IOP profile in a more feasible and controlled fashion.^[4,5]^

IOP changes after WDT have been evaluated in adult patients with various types of glaucoma,^[6,7]^ but not in children with PCG. Previous studies also evaluated the WDT-IOP profile of adult glaucoma patients who were taking glaucoma medications or had undergone trabeculectomy, deep sclerectomy, and GDD implantation.^[8,9,10,11]^ However, there is no study in patients with PCG with prior trabeculotomy or GDD implantation.

The main objective of the present study was to evaluate the IOP changes after WDT in patients with PCG and to compare the IOP changes in those with the history of trabeculotomy and those who underwent trabeculotomy followed by GDD surgery.

##  METHODS

This prospective interventional study was conducted in a tertiary eye care hospital after getting approval from the local Ethics Committee. The study followed the principles of the Declaration of Helsinki, and informed consent was obtained from the parents of all participants. All enrolled patients underwent a complete ophthalmological examination, which included checking visual acuity, IOP measurement, and a dilated stereoscopic fundus examination to assess the amount of optic nerve head damage using Disc Damage Likelihood Scale.^[12]^ Subsequently, those who met the eligibility criteria were included. The average thickness of the peripapillary retinal nerve fiber layer (using optical coherent tomography) and the central corneal thickness were also recorded.

At our center, all congenital glaucoma patients undergo trabeculotomy at the superonasal and inferotemporal sites in one session, and if the IOP cannot be controlled using medications, Ahmed Glaucoma Valve (FP7, New World Medical, Rancho Cucamonga, LA, USA) is implanted in the superotemporal quadrant. We do not perform trabeculectomy because of the high chance of failure. Therefore, all patients in the current study had the history of trabeculotomy procedure as the first line treatment. The inclusion criterion was having a controlled PCG with office IOP equal to or under 22 mmHg with or without medication. The exclusion criteria were the presence of ocular infection, corneal opacity, or scar preventing reliable IOP measurements; active heart or renal diseases; and refusal of parents to enroll their children in the study.

### Water drinking test

Subjects were instructed to refrain from food and fluid intake 3 hours preceding the WDT. After checking the baseline IOP, patients drank 15 mL/kg of bottled water in five minutes. Subsequently, IOP was measured every 15 min for 1 hour. The IOP was measured five times (baseline, 15, 30, 45, and 60 min after drinking water). One examiner measured the IOP using a non-contact tonometer (CT80; Topcon Co., Tokyo, Japan). The average of three measurements was recorded and the measurements were repeated if the differences between the three measurements were greater than 3 mmHg. The following parameters were assessed: IOP trough (lowest IOP after drinking water), IOP peak (highest IOP after drinking water), IOP mean (the mean of the four IOPs after drinking water), IOP fluctuation (difference between peak IOP and baseline), IOP range (difference between peak IOP and lowest IOP reading after drinking water), and end-pressure difference (IOP at 60 min versus baseline).

### Statistical analysis

The IOP of both eyes was measured, but one eye was randomly selected (using a randomization chart generated by a randomization software) for inclusion in the study. All data were analyzed using IBM SPSS Statistics software version 21 (SPSS Inc., Chicago, IL) and MedCalc version 12.2.1 (*MedCalc* Software, Mariakerke, Belgium). Descriptive results were presented as the mean ± standard deviation (SD). A *P*-value < 0.05 was considered to be statistically significant.

##  RESULTS

Patients' baseline characteristics are presented in Table 1. Of the 20 studied patients, 17 (85%) had no associated systemic disease. Cardiac disease (repaired ventricular septal defect), phenylketonuria, and glucose-6-phosphate dehydrogenase deficiency were each found in one patient. However, no subject was on systemic medications, and no patient was prohibited from undergoing the WDT by the pediatrician.

**Table 1:** Baseline characteristics of patients with primary congenital glaucoma


**Characteristic**	**Value**
**Age, year(s)**	9.9 ± 2.7 (6 to 16)${}^{a}$
**Gender, (Male/Female)**	8/12
**Eye, (Right/Left)**	12/8
**Weight, kg**	30.3 ± 12.2 (14 to 53)${}^{a}$
**Height, cm**	134 ± 17 (104 to 169)${}^{a}$
**Body Mass Index, kg/m${}^{2}$**	16.1 ± 3.4 (12.9 to 23.0)${}^{a}$
**Spherical Equivalent Refraction, Diopter(s)**	–2.7 ± 4.7 (–17.5 to +1.3)${}^{a}$
**Astigmatism, Diopter(s)**	–1.1 ± 1.1 (–4.5 to 0.0)${}^{a}$
**Central Corneal Thickness, µm**	582 ± 47 (488 to 653)${}^{a}$
**Cup-to-Disc ratio, %**	48 ± 24 (10 to 80)${}^{a}$
**Average Retinal Nerve Fiber Layer Thickness, µm**	92 ± 23 (51 to 140)${}^{a}$
**Number of Topical Medications**	1.4 ± 1.1 (0 to 3)${}^{a}$
**Lens status, ** ***n*** ** (%)**	Phakic: 20 (100)
**Operation, ** ***n*** ** (%)**	Trabeculotomy only: 14 (70) GDD 6 (30)
**Baseline Intraocular Pressure, mm Hg**	15.8 ± 3.7 (8.5 to 21.0)${}^{a}$
${}^{a}$Scalar data are presented as mean ± standard deviation (range)	

**Table 2:** Comparison of WDT-IOP parameters between the GDD (*n* = 6) and the trabeculotomy (*n* = 14) group


**Parameter (mmHg)**	**Operation**		**** ***P*** **-value${}^{b}$**
	**Trabeculotomy${}^{a}$**	**GDD${}^{a}$**	
**IOP Trough**	16.0 ± 2.9	16.7 ± 4.1	0.66
**IOP Peak**	20.1 ± 3.4	19.6 ± 4.1	0.75
**IOP Mean**	18.0 ± 3.2	18.3 ± 3.9	0.86
**IOP Fluctuation**	5.0 ± 2.6	2.5 ± 3.1	0.08
**IOP Range**	4.2 ± 1.8	2.9 ± 1.6	0.14
**End Pressure Difference**	1.5 ± 3.1	0.3 ± 2.9	0.41
${}^{a}$All data are presented as mean ± standard deviation; ${}^{b}$Calculated with Independent Samples *T*-test; all measurements passed the Shapiro–Wilk test of normality IOP, intraocular pressure; GDD, glaucoma drainage device; WDT, water drinking test			

**Table 3:** The results of the water drinking test in normal subjects, primary open angle glaucoma, ocular hypertension, and pseudoexfoliation syndrome


**Authors**	**Diagnosis**	**Management of glaucoma**	**Average age of patients (years)**	**IOP baseline (mmHg)**	**IOP peak (mmHg)**	**IOP fluctuation (mmHg)**
**Ozyol et al** ^[15]^	XFS (34)	No treatment	16.3	18.1	1.8
	XFG(30)	Medical therapy	65.6	19.7	26.9	7.2
**De Moraes et al** ^[16]^	POAG (22)	Medical therapy	54.3	12.4	20.00	7.6
**Danesh-Meyers et al** ${}^{\left[29\right]}$	POAG	Trabeculectomy	70	10.4	11.7	1.3
	Medical therapy	68	11.4	17.3	5.9
**Mansouri et al** ^[8]^	POAG	Trabeculectomy	67.1	10.1	12.5	2.4
	Deep Sclerectomy	72.5	13.8	17.6	3.8
	Latanoprost	71.2	15.9	21.1	5.2
**Mansouri et al** ^[14]^	Normal subjects (25)	No treatment	35.6	14.9	16.8	1.9
**Guedes RA et al** ^[10]^	Normal subjects (20)	No treatment	58.9	13.9	15.8	1.9
	Glaucoma subjects (21)	No treatment	17.5	26	8.4
	Glaucoma subjects (21)	Dorzolamide-timolol	14.2	18.6	4.3
	Glaucoma subjects (15)	Deep sclerectomy	12.3	14.1	1.7
	Glaucoma subjects (21)	Trabeculectomy	10	11.6	1.6
**Kocabeyoglu et al** ${}^{\left[30\right]}$	Normal subjects (20)	No treatment	64.4	14	15.5	1.5
	XFS (20)	No treatment	66.1	15	17.2	2.2
**Kerr et al** ^[13]^	POAG and OHTN	Before SLT	73	16.9	21	4.1
	After SLT	14.2	16.5	2.3
**Martinez et al** ^[11]^	POAG	Trabeculectomy (20)	67.9	12.3	16.25	3.95
	GDD (20)	66.2	12.5	16.15	3.6
XFS, pseudoexfoliation syndrome; XFG, pseudoexfoliative glaucoma; POAG, primary open angle glaucoma; OHTN, ocular hypertension; SLT, selective laser trabeculoplasty			

**Figure 1:**
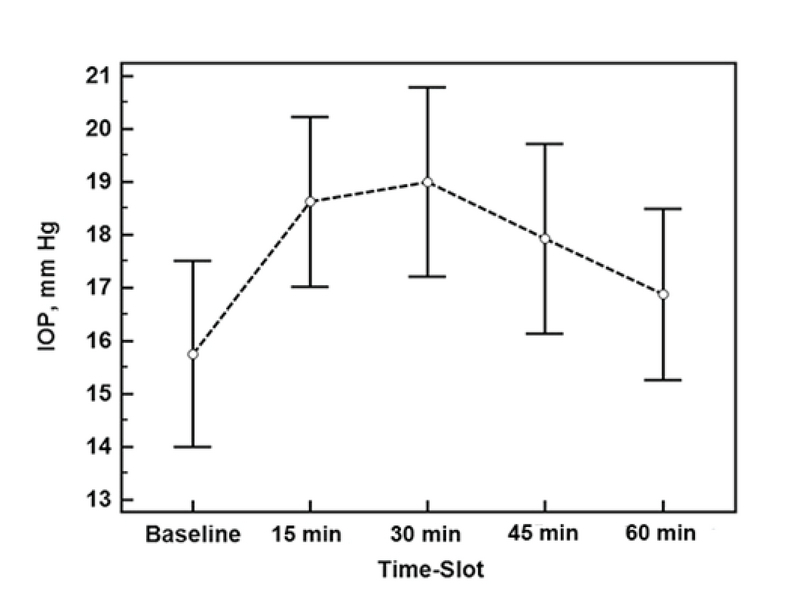
The average intraocular pressure at baseline and at each time-point after the water drinking test in patients with congenital glaucoma.

The mean IOPs at baseline, and 15, 30, 45, and 60 min after WDT were 15.8 ± 3.7, 18.6 ± 3.4, 19.0 ± 3.8, 17.9 ± 3.8, and 16.9 ± 3.5 mm Hg, respectively (*P* < 0.001, repeated-measures analysis of variance (ANOVA); Figure 1). Pairwise comparisons using Bonferroni correction revealed that the mean IOPs 15 and 30 min after WDT were significantly greater than the baseline IOP (*P* < 0.001 and *P* = 0.002, respectively), however, the mean IOPs after 45 and 60 min were not (*P* = 0.062 and *P* = 1, respectively). The IOP after 60 min was significantly lower than the IOP after 30 min (*P* = 0.03).

**Figure 2:**
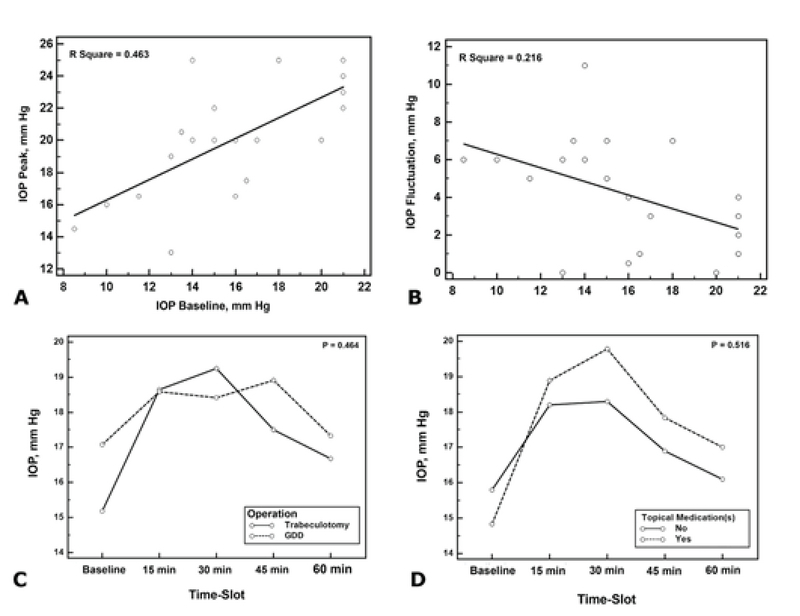
(A) The scatter diagram and regression line showing direct association between IOP baseline and IOP peak. (B) The scatter diagram and regression line showing reverse association between IOP baseline and IOP fluctuation. (C) The IOP profile after the water drinking test in the trabeculotomy and glaucoma drainage device implantation groups. (D) The IOP profile after the water drinking test in eyes that underwent trabeculotomy with and without adjunctive topical antiglaucoma medications.

The values of different WDT-IOP parameters were as following: IOP trough, 16.2 ± 3.2 (range, 10.0 to 22.0) mm Hg; IOP peak, 20.0 ± 3.5 (13.0 to 25.0); IOP mean, 18.1 ± 3.3 (12.0 to 23.3); IOP fluctuation, 4.2 ± 2.9 (0.0 to 11.0); IOP range, 3.8 ± 1.8 (1.0 to 7.0); and end-pressure difference, 1.1 ± 3.1 (–4.0 to 7.0). The first time-point to show an IOP peak was 15 min in nine patients (45%), 30 min in six (30%) patients, 45 min in three (15%) patients, and 60 min in two (10%) patients.

Linear regression analysis revealed the IOP baseline to be the only statistically significant determinant of the IOP peak (R${}^{2}$ = 0.463; *P* = 0.001; Figure 2A). The use of a higher number of topical medications was also associated with a trend toward higher IOP peak values (R${}^{2}$ = 0.170; *P* = 0.071). IOP fluctuation was significantly associated with the IOP baseline (R${}^{2}$ = 0.216; *P *= 0.039; Figure 2B); and it was lower in the GDD group compared with the trabeculotomy group (R${}^{2}$ = 0.158; *P* = 0.082).

Figure 2C and Table 2 summarize the results of WDT in the GDD group and trabeculotomy alone group. The repeated measures analysis of covariance (assuming age, gender, body mass index (BMI), and number of topical medications as possible covariates) revealed no significant difference in WDT-IOP changes between the two surgical groups (*P* = 0.46; Figure 2C). Similarly, with the exception of the IOP fluctuation, which was marginally greater in the trabeculotomy alone group than in the GDD group (5.0 vs 2.5 mm Hg; *P* = 0.08; Table 2), the WDT-IOP parameters were not significantly different. However, because of the small sample size of the groups, the possibility of a type 2 error should be considered while interpreting the insignificant *P*-values.

Figure 2D shows the WDT-IOP changes in eyes that underwent trabeculotomy with and without adjunctive topical antiglaucoma medications.

##  DISCUSSION

Previous studies evaluated the WDT response in medically treated glaucoma and in adults who underwent trabeculectomy or GDD implantation.^[9,11]^ In our study involving PCG patients, the mean IOPs 15 and 30 min, but not 45 and 60 min, after WDT were significantly greater than the baseline IOP. The highest mean IOP was observed after 30 min. In the study by Martinez et al,^[11]^ comparing the results of the WDT in 40 eyes of 34 primary open angle glaucoma (POAG) patients who underwent trabeculectomy or GDD implantation, the highest mean IOP in both groups was observed 30 min after WDT. Similarly, 20 eyes from 20 POAG or ocular hypertension patients had the highest mean IOP after WDT 30 min following selective laser trabeculoplasty; however, before the laser procedure the highest mean IOP was observed after 45 min.^[13]^ In the study by Mansouri et al^[14]^ involving normal subjects, the highest mean IOP was detected 15 min after the WDT. The ability of the outflow pathway to handle the load after WDT may have affected the time at which the highest mean IOP was detected. In normal adults with normal outflow facility, the highest IOP was observed after 15 min; however, in adult patients who underwent trabeculectomy or GDD implantation and in our patients, the highest IOP was observed after 30 min.^[11,14]^

In our study the IOP fluctuation in all patients (GDD plus trabeculectomy), and in each of the trabeculectomy, and GDD groups were 4.2, 5.0, and 2.5 mmHg, respectively. The reported IOP fluctuation in adult glaucoma patients managed medically ranged from 4.3 to 8.4 mmHg.^[8,9,10,15,16]^ The reported IOP fluctuations in eyes that underwent trabeculectomy ranged from 1.6 to 3.95 (Table 3).^[8,10,11]^

A study on GDDs reported an IOP fluctuation of 3.6 mmHg.^[11]^ The IOP fluctuation in our trabeculotomy group (4.2 mmHg) was greater than the values reported in trabeculectomy (3.95 mmHg) and GDD (3.6 mmHg) groups in previous studies. However, the IOP fluctuation in our GDD group (2.5 mmHg) was lower than the value in POAG patients with GDD (3.6 mmHg).^[11]^

Several studies have suggested that IOP fluctuation is an important contributor to the risk of glaucoma progression.^[8,17]^ The Early Manifest Glaucoma Trial showed that even a 1 mmHg increase in IOP was associated with an 11% increase in the hazard ratio for glaucoma progression.^[18]^ The Advanced Glaucoma Intervention Study Group suggested that IOP peaks should be below 18 mmHg to prevent visual-field deterioration in patients with moderate- or advanced-stage glaucoma.^[19]^ As glaucoma progression is correlated with IOP peaks and fluctuations,^[20]^ accurate identification of at-risk patients has become imperative as the first step in preventing further irreversible glaucomatous damage.^[21]^ It has been shown that, in two-thirds of glaucoma patients, the highest IOP values occur outside regular clinic hours, frequently during the nocturnal/sleep period.^[22]^ Therefore, significant IOP fluctuation may be missed if relying only on clinic IOP measurements. Twenty-four hour IOP monitoring and provocative tests such as WDT were suggested as viable options for identifying a greater number of patients with poorly controlled glaucoma. A group of normal tension glaucoma patients underwent several clinical tests for predicting the progression of visual field loss, and the WDT was the most useful clinical predictor for visual field progression.^[5]^ The IOP peak occurred during home tonometry in approximately 30% of patients with progressive visual field loss while it occurred during home tonometry in 5% of patients with stable visual fields.^[23]^ After drinking water, the ability of the outflow system to modulate the stress of an IOP rise is the only mechanism that can control the IOP. Interventions that improve outflow facility can be expected to induce fewer changes in the IOP after WDT.

The smoother WDT-IOP profile in our GDD group may have a protective effect on the damaged optic nerve. It is plausible that trabeculotomy increases aqueous outflow, but not as effective as GDD surgery, which bypasses the congenitally abnormal aqueous drainage pathway in PCG. The IOP fluctuation in the trabeculotomy group was two times greater than that in the GDD group (5.0 vs 2.5 mmHg; *P* = 0.08).

The IOP profile in the trabeculotomy group on glaucoma medications was greater, though not statistically significant, compared to the trabeculotomy group who were not on medication (Figure 2D). A trend toward a greater IOP peak was observed as the number of topical medications increased (R${}^{2}$ = 0.170; *P* = 0.071). The use of glaucoma medications following the surgical procedure indicates insufficient IOP control and suggests the existence of increased resistance to the outflow. In other words, the higher number of medications may be an indirect measure of the increased resistance in the outflow pathway.

The baseline IOP was the only significant determinant of the IOP peak (R${}^{2}$ = 0.463; *P* = 0.001). This is in line with the findings of previous studies in adult patients demonstrating that a higher IOP at baseline is associated with greater 24-hour IOP changes when measured in the seated position.^[24]^ The rate of aqueous production is steady and the outflow facility is the only determinant factor of IOP. When the baseline IOP is low, the possibility of IOP fluctuation might be reduced because the outflow pathway can handle the load and vice versa.

IOP variation over time may be divided into diurnal, short-term, and long-term fluctuations. It is often difficult to get a true picture of a patient's IOP profile when it is measured only several times a year. The current method of IOP measurements is simply a snapshot of the real IOP over time and does not represent the actual IOP profile. The WDT is utilized as a provocative test to evaluate outflow capacitance and the effect of medical or surgical glaucoma treatments on the IOP peak and fluctuation.^[19]^ Studies have shown that the WDT-IOP peak strongly correlates with the peak of shortened diurnal curves and the long-term IOP profile.^[6,16]^ The exact mechanism that underlies IOP elevation after water ingestion remains unclear. The proposed mechanisms include choroidal expansion, plasma hypo-osmolality-enhanced aqueous ultrafiltration, autonomic nervous system stimulation, and increased episcleral venous pressure.^[7,19]^ Compared to the 24-hour IOP curve measurement that requires the patient to stay in the hospital and involves the measurement of IOPs at night, the WDT could be an inexpensive and feasible alternative.

This study has several limitations including the small number of patients, especially in the GDD group, and the fact that the IOP was measured using an air-puff tonometer. In most studies that involved performing WDT in adult glaucoma patients, the number of participants was around 20–30 patients, and in some studies both eyes were included (Table 3). In this study, we included one eye from each patient. The global prevalence of glaucoma for the population aged 40–80 years is 3.54%, which is much greater than that for PCG (0.01–0.001%).^[25,26]^ The rarity of this disease makes recruiting PCG patients challenging. With respect to cooperation for IOP measurement, non-Goldmann tonometer are usually used for IOP measurement in pediatric patients. It has been shown that, in PCG patients, IOP values obtained using an air-puff tonometer are similar to those obtained using a Goldmann tonometer.^[27]^ Additionally, in a recent meta-analysis that compared all available tonometers with the Goldmann applanation tonometer, air-puff tonometers yielded the least amount of variability in IOP values (mean difference of 0.2 mm Hg).^[28]^

In conclusion, the WDT induced significant IOP elevation 15 and 30 min after the test in patients with PCG. This increased IOP returned to pre-test values after 45 min. In eyes previously treated with trabeculotomy, the IOP fluctuation was greater, though not statistically significant.

##  Financial Support and Sponsorship

Nil.

##  Conflicts of Interest

There are no conflicts of interest.
